# Primed to comply: Individual participant data sharing statements on ClinicalTrials.gov

**DOI:** 10.1371/journal.pone.0226143

**Published:** 2020-02-18

**Authors:** Emily E. Statham, Sarah A. White, Bhagyashree Sonwane, Barbara E. Bierer

**Affiliations:** 1 The Multi-Regional Clinical Trials Center of Brigham and Women’s Hospital and Harvard, Cambridge, Massachusetts, United States of America; 2 Department of Medicine, Division of Global Health Equity, Brigham and Women’s Hospital, Boston, Massachusetts Harvard Medical School, Boston, Massachussetts, United States of America; 3 Harvard Medical School, Boston, Massachussetts, United States of America; University Medical Center Utrecht, NETHERLANDS

## Abstract

In June 2017, the International Committee of Medical Journal Editors (ICMJE) announced a requirement that authors reporting the results of clinical trials to journals that follow ICMJE recommendations must include an individual participant data (IPD) sharing statement with manuscripts submitted after 01 July 2018. Additionally, all new clinical trials for which enrollment began on or after 01 January 2019 must include a data sharing statement in the trial’s publicly posted registration. This study sought to understand whether IPD sharing statements of clinical trials first registered on ClinicalTrials.gov before 01 January 2019 reflected comprehension of the expectations and a willingness to share. To establish baseline characteristics for the prevalence and quality of IPD sharing statements, we examined IPD sharing statements among 2,040 clinical trials first posted on ClinicalTrials.gov between 01 January 2018 and 06 June 2018. Two independent coders further analyzed the quality of the IPD sharing statements of trials whose registration records indicated the intent to share IPD. The vast majority of trials included in this study did not indicate an intent to share IPD (n = 1,928; 94.5%). Among the trials that did commit to sharing IPD (n = 112, 5.5%), significant variability existed in the content and structure of IPD sharing statements. The results of this study suggest that successful compliance with the IPD sharing statement requirements of the ICMJE will require further clarification, enhanced education, and outreach to investigators.

## Introduction

In June of 2017, the International Committee of Medical Journal Editors (ICMJE) introduced new requirements for the publication of clinical trial reports in ICMJE journals [1]. Effective 01 July 2018, the new ICMJE requirements mandated that authors reporting the results of clinical trials to journals that follow ICMJE recommendations must include a data sharing statement at the time of manuscript submission. In addition, all new clinical trials for which enrollment began on or after 01 January 2019 must include a data sharing statement in the trial’s publicly posted registration [1]. Importantly, the ICMJE requirement does not require that this data sharing statement reflect an intent to share IPD. Instead, it only requires transparency with respect to whether and under what conditions the investigators plan to share IPD.

The ICMJE requirements came as no surprise. In April 2015, the Institute of Medicine published a consensus report entitled “Sharing Clinical Trial Data: Maximizing Benefits, Minimizing Risk.” The committee recommended guiding principles and a framework for sharing clinical trial data that addressed what data should be shared, when, and under what conditions [2]. Shortly thereafter in January of 2016, the ICMJE requested public feedback on the proposal entitled “Sharing Clinical Trial Data: A Proposal From the International Committee of Medical Journal Editors” to require authors not only to share individual participant data (IPD) with others but also to include a plan for data sharing at trial registration [3]. Citing practical challenges to implementation and perceived risks of IPD sharing, the 2017 ICMJE requirement required a data sharing statement at the time of manuscript submission for all manuscripts submitted after 01 July 2018 and, later, a data sharing plan posted at the time of trial registration for all manuscripts submitted after 01 January 2019. The ICMJE provided detailed instructions for the data sharing plan and adequate notice prior to the effective dates for compliance [1].

In the 2016 editorial, the ICMJE recognizes IPD as de-identified individual participant level data and measurements including tables, figures, and appendices or supplementary material that support the results presented in a manuscript [3]. Of note, this is different than the aggregate data submitted to ClinicalTrials.gov Protocol Registration and Results System (PRS). According to the ICMJE, data sharing statements that reflect the intent to share IPD must address specific informational elements: “whether individual deidentified participant data (including data dictionaries) will be shared; whether additional, related documents will be available (e.g., study protocol, statistical analysis plan, etc.); when the data will become available and for how long; by what access criteria data will be shared (including with whom, for what types of analyses, and by what mechanism).” [1] Only data sharing statements that reflect the intent to share IPD are required to provide such information. The ICMJE generated four examples of compliant data sharing statements, each of which embodies a distinct approach to IPD sharing [1]. Three of the four examples of data sharing statements indicate that deidentified IPD will be shared, describe the particular data that will be shared, the additional documents that will be provided, give the start and end dates within which IPD will be made available, provide the webpage URL whereby the IPD may be requested, and specify the terms of access to the shared IPD. The forth example indicates that IPD will not be shared.

In December 2015, ClinicalTrials.gov added 2 optional registration fields to the PRS to address IPD Sharing: “Plan to Share IPD” and “Available IPD/Information Type” [4]. Based on an August 2017 analysis conducted by Bergeris et. al. [4] that found potential confusion in the meaning of the terms IPD and sharing, and the inconsistent responses found in their qualitative analysis, ClinicalTrials.gov added additional subfields to the IPD Sharing Statement section in late June 2017 [4,5]. The current IPD sharing registration fields in the ClinicalTrials.gov PRS include: “Plan to Share IPD”, “Plan Description, “IPD Sharing,” “Supporting Information,” “Time Frame,” “Access Criteria,” and “URL.” Definitions of these IPD sharing terms and specific instructions are available on the ClinicalTrials.gov website [5].

In order to evaluate the impact of the ICMJE requirements on the inclusion of data sharing statements at the time of trial registration, we sought to establish baseline characteristics for the frequency and quality of these statements. We therefore evaluated IPD sharing statements among 2,040 clinical trials that were first posted on ClinicalTrials.gov between 01 January 2018 and 06 June 2018. This represents a 5 month period of time after publication of the ICMJE requirements (June 2017) and the subsequent addition of registration subfields to provide clarity about IPD sharing by ClinicalTrials.gov (June 2017) [4] but before the effective date of the ICMJE requirement requiring that data sharing statements be included at the time of manuscript submission (July 2018). We also determined whether and to what degree the available data sharing statements fulfilled the ICMJE requirements in order to identify which elements of these requirements might benefit from additional clarification.

## Materials and methods

Using data elements identified through the ClinicalTrials.gov PRS, we requested a custom report for trials first posted on ClinicalTrials.gov between 01 January 2018 and 06 June 2018. The selection criteria of the custom report limited the trials to active interventional studies with a recruitment status of “Recruiting,” “Enrolling by Invitation,” or “Active, but not Enrolling” and that had at least one location in the United States. ClinicalTrials.gov generated the custom report on 22 June 2018. A total of 2,040 studies met the selection criteria specified above. S1 Table provides the output data elements of the custom report.

The comma-separated values (CSV) files of all 2,040 study records were downloaded on 22 June 2018; data elements were mapped into an excel-readable format for further analysis. The report output data elements of several ClinicalTrials.gov registration fields (S2 Table) were coded independently into aggregated categories by two coders (ES and BS). The inter-rater reliability was 98.4%. Discrepancies were discussed among the two coders until consensus was reached. The incidence of IPD sharing was then calculated with respect to trials’ recruitment status, lead sponsor, number of funders, involvement of an FDA-regulated drug or device, primary study purpose, study phase, and enrollment size.

Those studies that indicated an intent to share IPD by selecting “Yes” under the optional protocol registration field entitled “IPD Sharing” (n = 112 studies) were further coded independently by the same two coders (ES and BS). The inter-rater reliability was 100%. The coded outcome data elements were further defined by one reviewer (ES).

## Results

### Intent to share IPD

During the five months from 01 January 2018 to 06 June 2018, 2,040 trials met the selection criteria of active, interventional studies with at least one location in the United States and were posted to ClinicalTrials.gov. The study characteristics including recruitment and enrollment status, sponsor type, study phase, and enrollment count are shown in Table 1. Of 2,040 studies, 112 (5.5%) reported “Yes” under “IPD sharing;” 1,928 (94.5%) reported “Not Yes.” Notably, the percentage of studies that do or do not plan to share IPD was found to be similar regardless of the study characteristic against which it was measured (Table 1). Of the fields that were completed (not left blank), whether the frequency of IPD sharing was determined on the basis of trials’ recruitment status, lead sponsor, number of funders, involvement of an FDA-regulated drug or device, primary study purpose, study phase, or enrollment count, the average percentage of studies that selected “Yes” under “IPD Sharing” was 6.1% and ranged from 3.8% to 11.9%.

**Table 1:** Studies’ intent to share IPD by coded sub-category of study characteristic.

Study Characteristic	Coded Sub-Categories (number of studies out of 2,040 total studies)	Number of Studies That Plan to Share IPD Per Sub-Category	
		Yes (N = 112)	Not Yes (N = 1,928)
**Recruitment Status**	Active, Not Recruiting (n = 107)	7/107 (6.5%)	100/107 (93.5%)
	Enrolling by Invitation (n = 138)	10/138 (7.2%)	128/138 (92.8%)
	Recruiting (n = 1,795)	95/1,795 (5.3%)	1,700/1,795 (94.7%)
**Lead Sponsor**	Academia (n = 1,322)	68/1,322 (5.1%)	1,254/1,322 (94.9%)
	Industry (n = 638)	39/638 (6.1%)	599/638 (93.9%)
	Federal Agency (n = 80)	5/80 (6.2%)	75/80 (93.8%)
**Number of Funders**	Single Funder (n = 1,579)	87/1,579 (5.5%)	1,492/1,579 (94.5%)
	Multiple Funder (n = 461)	25/461 (5.4%)	436/461 (94.6%)
**Involvement of FDA Regulated Drug or Device**	Yes (n = 1,224)	61/1,224 (5%)	1,163/1,224 (95%)
	No (n = 802)	51/802 (6.4%)	751/802 (93.6%)
	Field Left Blank (n = 14)	0/14 (0%)	14/14 (100%)
**Primary Study Purpose**	Treatment (n = 1,198)	58/1,198 (4.8%)	1,140/1,198 (95.2%)
	Not Treatment (n = 842)	54/842 (6.4%)	788/842 (93.6%)
**Study Phase**	Early Phase (1, 1|2, 2) (n = 733)	28/733 (3.8%)	705/733 (96.2%)
	Late Phase (2|3, 3, 4) (n = 299)	30/299 (10%)	269/299 (90%)
	Field Left Blank (n = 1,008)	54/1,008 (5.4%)	954/1,008 (94.6%)
**Enrollment Count**	Small (1–50) (n = 891)	40/891 (4.5%)	851/891 (95.5%)
	Medium (51–100) (n = 436)	17/436 (3.9%)	419/436 (96.1%)
	Large (101–500) (n = 554)	36/554 (6.5%)	518/554 (93.5%)
	Extra Large (500+) (n = 159)	19/159 (11.9%)	140/159 (88.1%)

### IPD sharing statements

Further qualitative analysis was conducted on the studies that indicated an intent to share IPD (n = 112 studies). Of 112 studies, 6 (5.4%) left the field entitled “IPD Sharing Description” blank and 106 (94.6%) included a free-text IPD-sharing description (Table 2). Of the 106 studies that included a description, 7 (6.6%) described general dissemination and publication strategies, not IPD sharing. Four (3.8%) studies described sharing research data among team members (e.g. “Only researchers listed in the protocol will have access to IPD,” and “De-identified data will be available to the local site PIs”), and did not describe IPD sharing. Ninety-five (89.6%) of the 112 studies provided a response that addressed IPD sharing.

**Table 2:** Content of IPD sharing statements.

ClinicalTrials.gov Registration Field	Coded Outcome Data Elements	Number of Studies Per Coded Outcome Data Element	Defined Outcome Data Elements	Number of Studies Per Coded Outcome Data Element
IPD Sharing Description	Response Provided	106/112 (94.6%)	General dissemination and publication strategies, not IPD sharing	7/106 (6.6%)
			Sharing data among research team, not IPD Sharing	4/106 (3.8%)
			Response addressed IPD sharing	95/106 (89.6%)
	Blank	6/112 (5.4%)	n/a	n/a
IPD Sharing Information Type	Analytic Code	1/112 (0.9%)	n/a	n/a
	Clinical Study Report (CSR)	3/112 (2.7%)	n/a	n/a
	Statistical Analysis Plan (SAP)	3/112 (2.7%)	n/a	n/a
	Study Protocol	56/112 (50%)	n/a	n/a
	Blank	49/112 (43.7%)	n/a	n/a
IPD Sharing Time Frame	Response Provided	67/112 (59.8%)	Insufficient or unclear	8/67 (11.9%)
			IPD sharing upon request/ to be determined	10/67 (14.9%)
			General or ambiguous time frame	12/67 (17.9%)
			Only start date specified	19/67 (28.4%)
			Start and end dates specified	18/67 (26.9%)
	Blank	45/112 (40.2%)	n/a	n/a
IPD Sharing Access Criteria	Free Text	61/112 (54.5%)	Unclear or insufficient entry	11/61 (18%)
			Access criteria not yet determined	3/61 (4.9%)
			Entry does not address all 3 informational requirements	44/61 (72.1%)
			Entry addresses all 3 informational requirements	3/61 (4.9%)
	Blank	51/112 (45.5%)	n/a	n/a
IPD Sharing URL	URL Provided	18/112 (16.1%)	Direct link to webpage where data may be requested	13/18 (72.2%)
			Direct link to relevant data sharing requirement	3/18 (16.7%)
			Link broken	2/18 (11.1%)
	Blank	94/112 (83.9%)	n/a	n/a

Of the 112 studies, 63 (56.3%) completed the field “IPD Sharing Information Type” that listed what supporting information or other documents would be shared in addition to the IPD; Forty-nine (43.7%) did not provide a response. Of these 112 studies, 56 (50%) planned to share the study protocol; 3 (2.7%) planned to share the clinical study report (CSR); 3 (2.7%) planned to share the statistical analysis plan (SAP); and 1 (0.9%) planned to share analytic code (Table 2).

Analysis of the field “IPD Sharing Time Frame” showed that of 112 studies, 67 (59.8%) included a free-text entry. Eight (11.9%) of these entries contained information that was coded as insufficient or unclear and an additional 10 (14.9%) featured some variation of the phrase “upon request,” or “to be determined,” addressing neither when the IPD would become available nor for how long investigators would have access to it (Table 2). Twelve (17.9%) of the 67 entries featured general or ambiguous time frames within which IPD would be made accessible to secondary researchers, such as, “at time of completion until the parent study concludes.” Nineteen entries (28.4%) specified only the start date but not the end date of IPD sharing. Only 18 (26.9%) of the 67 entries provided estimates of both the start and end dates (Table 2).

The majority (n = 61; 54.5%) of 112 completed the registration field entitled “IPD Sharing Access Criteria.” Of these 61 studies, only 3 (4.9%) entries addressed all three of the ICMJE-recommended informational elements that are pertinent to this field: with whom data will be shared, for what types of analyses the data will be made available, and by what mechanism the data will be made available. Three of the 61 studies (4.9%) stated that the IPD sharing access criteria were not fully developed and/or will be determined at a later time, and 11 (18%) entries were coded as unresponsive, as they failed to contain information about access (e.g. “when we are ready to publish, the data will be available).” None of the remaining 44 (72.1%) entries addressed all three of the aforementioned ICMJE-recommended informational elements (Table 2).

Finally, 94 (83.9%) of 112 studies did not include a URL web address or other information to find additional information about requesting and accessing data, while 18 studies (16.1%) did **(**Table 2). Thirteen (72.2%) of the 18 URLs linked directly to the web page wherein data could be requested. Three (16.7%) of the 18 URLs linked to data sharing policies, and 2 (11.1%) of the URLs were broken.

## Discussion

To establish a baseline understanding of the clinical trial data sharing landscape, we assessed the prevalence and characteristics of data sharing statements first posted on ClinicalTrials.gov during a period of time after the publication of the ICMJE data sharing statement requirements and availability of the current IPD Sharing data elements in ClinicalTrials.gov and before the effective date of 01 July 2018 wherein data sharing statements were required at the time of manuscript submission. In other words, the responsible parties voluntarily completed the data sharing fields in ClinicalTrials.gov at a time after the guidance and explanatory text became available.

The vast majority of the 2,040 trials included in our analysis (n = 1,928; 94.5%) did not indicate an intent to share IPD. Of the 112 studies that did indicate an intent to share IPD, analysis revealed uncertainty in or insufficient understanding of the definition of IPD and the process of IPD sharing. Our findings are consistent with the 2018 Bergeris et. al. editorial that found “confusion or uncertainty about the term IPD and the meaning of the term sharing” [4]. This “confusion or uncertainty” appeared to persist despite the addition of additional subfields and explanation provided on ClinicalTrials.gov in response to the findings of Bergeris and co-authors.

While we choose a sample of studies registered after the ICMJE requirements were published in June of 2017, the the ICMJE requirements were not yet effective. Therefore, the small minority of trials that did indicate an intent to share IPD may not capture or reflect all that will. It is possible that study sponsors were aware of the requirements but chose not to identify intent to share or the details of the IPD sharing plans until required either at manuscript submission (effective July 2018) or at the initial registration in the ClinicalTrials.gov PRS for new studies beginning in January 2019. Additionally, for example, many large pharmaceutical companies have internal data sharing policies that only go into effect once a trial is complete. It is therefore possible that some of the trials whose ClinicalTrials.gov registration records indicated no intent to share IPD will, in fact, make data available for sharing later through company-specific efforts.

The analysis of data presented here suggests that the subfields and structure added to ClinicalTrials.gov in June 2017 are insufficient; successful compliance with the ICMJE IPD sharing statement requirements could be facilitated by further clarification and education about IPD sharing as well as the intent of the requirements. We offer three recommendations to foster data sharing.

1. Provide tools to facilitate clarification of the ICMJE requirements on ClinicalTrials.gov: Presently, the ClinicalTrials.gov Protocol Registration Data Element Definitions for Interventional and Observational Studies document provides definitions for the protocol registration fields, including the IPD Sharing fields [5] and definitions can be accessed by hyperlink within the specific registration modules. Based on our analysis, however, confusion and inconsistency exists regarding IPD sharing plans as defined in the ICMJE data sharing statement requirements. This may be due to lack of understanding of the IPD data sharing definitions or confusion between linking the ICMJE requirements with the corresponding optional protocol registration fields in ClinicalTrials.gov. In order to facilitate consistency in the format and informational contents of IPD sharing statements, additional detailed instructions on ClinicalTrials.gov would be useful. Furthermore, to reduce variability and enhance the overall quality and utility of IPD sharing descriptions, we propose a set of recommended instructions in Table 3 that can be used in organization and/or institutional guidance. The set of recommended instructions in Table 3 incorporates the ClinicalTrials.gov data element definitions and provides additional instructions and examples.
  In addition to providing instructional detail, developing additional educational resources and tools related to the generation of ICMJE-compliant IPD sharing statements may enhance consistency and utility of IPD sharing statements. The Stanford Center for Clinical and Translational Research and Education has created one such resource that maps the ICMJE-required informational elements of data sharing statements to the corresponding data elements of the ClinicalTrials.gov PRS [6]. Resources that bridge the ICMJE requirement with the practical steps to be taken by investigators are helpful.
2. Align expectations of IPD Sharing Plans across Grant Applications, Study Protocols, and ICMJE journals: Responsible IPD sharing is a process that requires investigators to integrate data sharing activities into all phases of the research lifecycle—from pre-trial funding applications and protocol development to post-trial dataset anonymization and data package preparation [7]. The requirement to provide IPD sharing statements, specifically, now exists at multiple points within this lifecycle, including at the time of certain grant applications, at the time of trial registration, and at the time of manuscript submission to ICMJE member journals [7]. The detail of the daring sharing responses analyzed in our work and presented in Table 2 indicate that unclear and inconsistent responses appear within the categories (i.e. sharing time frame and access criteria); different expectations may contribute to confusion.
  Generating high-quality IPD sharing statements requires time and thoughtful consideration on the part of sponsors and investigators; the administrative burdens of IPD sharing would be reduced by aligning data sharing statement requirements across funders, regulators, clinical trial protocols, and the ICMJE, among others. Streamlining these requirements would allow sponsors and investigators to generate one comprehensive IPD sharing statement that could be submitted, uploaded, and/or referenced as required. Moreover, were ClinicalTrials.gov to become the central location for posting data sharing plans, requests for data sharing statements could simply be referred to the site. Any change in plans after submission could be recorded in one location, again saving time and effort for investigators and sponsors.
3. Modify expectations of sponsors and investigators who do not intend to share IPD: Our analysis identified that 1,928 of 2040 studies (94.5%) reported “Not Yes” under “IPD sharing;” no further information or explanation is required. Under the ICMJE requirements for IPD sharing statements, investigators who intend to share IPD suffer a far greater administrative burden than those who do not: specifically, fewer tasks are associated with selecting “No,” “Undecided,” or leaving the field blank than selecting “Yes.” A “no” response requires no further action; no insight as to the reasons for declining data sharing is possible. Amending the ICMJE IPD sharing statement requirements such that investigators who do *not* intend to share IPD are encouraged or required to provide an explanation for the declination would be beneficial; like affirmative data sharing statements, that explanation would be useful at the time of manuscript review. The ICMJE points out that editors may take the intent to share IPD into account when making editorial decisions [1], and the ClinicalTrials.gov registration record would provide an explanation of the decision. Further, analysis of the reasons for declining might provide insight into other challenges or barriers to data sharing.

**Table 3:** Recommended instructions for completing the optional IPD sharing-specific protocol registration fields of the ClinicalTrials.gov PRS.

Informational Element of IPD Sharing Statements Required by the ICMJE	Corresponding Optional Protocol Registration Field in ClinicalTrials.gov	Recommended Instructions
Will IPD be available? (including data dictionaries)	**Plan to Share IPD**	Individual Participant Data (IPD) consist of raw participant data and/or cleaned, anonymized, and analyzable datasets. Based on this definition, please indicate whether there is a plan to make individual participant data (IPD) available to other researchers outside the current study protocol (i.e. secondary researchers).
		Select one: Yes, No, Undecided
		Note: ICMJE requires a Yes/No answer; Undecided is not acceptable.
What data in particular will be shared?	**Plan Description**	If you answered “Yes,” please provide a statement that includes the following information:
		Specific data that will be shared (i.e., de-identified IPD that underlies the results reported in the publication) When data sharing will begin and end (i.e., after publication of primary results; available indefinitely) To whom data will be made available (i.e., investigators whose proposed research has received IRB approval) How data will be made available (i.e., via a data repository following execution of data use agreement)
		If you answered “No,” please briefly describe why you are unable to share IPD from this study.
What other documents will be available?	**Supporting Information**	In addition to the IPD and data dictionaries, please select the type(s) of supporting information that will be shared. Select all that apply.
		Study Protocol Statistical Analysis Plan (SAP) Informed Consent Form (ICF) Clinical Study Report (CSR) Analytic Code Any other (If applicable)
When will data be available? (start and end date)	**Time Frame**	Please describe when the IPD and supporting information will become available to secondary researchers.
		Please indicate the period of availability.
		Your response may be formatted as an absolute date (i.e., beginning in January 2018 and available until December 2019), a date relative to another milestone (i.e., from the time of publication and for 2 years thereafter), or a period of time without exact dates (i.e., from the time of study completion and available indefinitely).
With whom? For what type of analyses? By what mechanism will data be made available?	**Access Criteria**	Please explain the mechanism by which IPD and supporting information will be made available. Please address the following elements:
		Who is eligible to access the data (i.e., qualified researchers who have received IRB approval) The process for reviewing and granting requests How data will be made available (i.e., via a repository after execution of a data use agreement)
	**URL**	Please share all web addresses that contain additional information about the plan to share IPD. Entries may include, but are not excluded to, the webpage for the repository in which the IPD will be deposited or the webpage that explains the eligibility criteria and internal review processes for reviewing data requests.

## Conclusion

The IPD sharing statements of 2,040 clinical trials first posted on ClinicalTrials.gov from 01 January 2018 to 06 June 2018 were analyzed in order to establish the baseline prevalence and quality of IPD sharing. The majority of trials included in this study did not indicate an intent to share IPD (n = 1,928; 94.5%). Among the trials that did (n = 112, 5.5%), significant variability was observed with respect to the specificity, clarity, and relevance of information provided in the data sharing statements. Several study sponsors described general dissemination and publication strategies and not IPD sharing; further, the informational elements of data sharing statements required by the ICMJE were rarely addressed in full.

These findings, consistent with Bergeris et. al. [4], suggest that the understanding of IPD sharing expectations is inadequate, even among investigators who intend to share IPD. With its data sharing statement requirement, the ICMJE has taken a meaningful step in advancing a culture of data sharing and data transparency in clinical research. For this requirement to have its intended impact, however, investigators would benefit from additional clarification on the required components of data sharing statements and the process by which detailed information should be included at the time of trial registration.

Increasing the prevalence of data sharing in clinical trials will require action from a number of stakeholder groups, including academic institutions, regulatory agencies, sponsors, funders, trial registries, journals, and investigators. By outlining several areas of additional clarification and future work, we hope to foster an environment wherein the sharing of IPD is feasible, executed with minimum administrative burden, aligned across and supported by all stakeholder groups within the clinical research community.

## Supporting information

S1 Table Outcome data elements collected for studies included in ClinicalTrials.gov custom report. (DOCX)

S2 Table Coding schema for ClinicalTrials.gov outcome elements. (DOCX)
