# Supplementary material for: Primed to comply: Individual participant data sharing statements on ClinicalTrials.gov
Source: PLoS One. 2020 Feb 18;15(2):e0226143. doi: 10.1371/journal.pone.0226143 (PMC7028256; doi:10.1371/journal.pone.0226143)
Supplement: S1 Table — (DOCX) [file pone.0226143.s001.docx]

**Table S1: Outcome Data Elements Collected for Studies Included in ClinicalTrials.gov Custom Report**

| Rank | NCT ID | Brief Title | Overall Status | Start Date | Primary Completion Date | Lead Sponsor |
| --- | --- | --- | --- | --- | --- | --- |
| Funded By | Responsible Party Name | Collaborator Names | Is FDA Regulated Drug | Is FDA Regulated Device | Is Unapproved Device | Conditions |
| Study Type | Primary Purposes | Phases | Enrollment Count | Minimum Age | Maximum Age | Gender |
| Number of Locations | Number of Unique Locations | Locations | IPD Sharing | IPD Sharing Description | IPD Sharing Info Type | IPD Sharing Time Frame |
| IPD Sharing Access Criteria | IPD Sharing URL | Available IPD Type | Available IPD Comment | Available IPD URL | Uploaded PDF | Document URLs |
