# Supplementary material for: Primed to comply: Individual participant data sharing statements on ClinicalTrials.gov
Source: PLoS One. 2020 Feb 18;15(2):e0226143. doi: 10.1371/journal.pone.0226143 (PMC7028256; doi:10.1371/journal.pone.0226143)
Supplement: S2 Table — (DOCX) [file pone.0226143.s002.docx]

**Supplemental Table 2: Coding Schema for ClinicalTrials.gov Outcome Elements**

| **ClinicalTrials.gov Registration Field** | **ClinicalTrials.gov Outcome Elements** | **Coded Outcome Elements** |
| --- | --- | --- |
| Overall Status | Active, Not Recruiting | Active, Not Recruiting |
|  | Enrolling by Invitation | Enrolling by Invitation |
|  | Recruiting | Recruiting |
| Lead Sponsor | Federal | Federal Agency |
|  | NIH |  |
|  | Other _ Government |  |
|  | Industry | Industry |
|  | Network | Academia |
|  | Individual |  |
|  | Other |  |
| Funded By | Federal | Single Funder |
|  | NIH |  |
|  | Industry |  |
|  | Other |  |
|  | Federal \| Industry | Multiple Funders |
|  | Federal \| Industry \| Other |  |
|  | Federal \| Other |  |
|  | Industry \| Other |  |
|  | NIH \| Federal \| Other |  |
|  | NIH \| Industry |  |
|  | NIH \| Industry \| Other |  |
|  | NIH \| Other |  |
| Is FDA Regulated Drug or Device | Is FDA Regulated Drug: YES | Yes |
|  | Is FDA Regulated Device: YES |  |
|  | Is FDA Regulated Drug: NO | No |
|  | Is FDA Regulated Device: NO |  |
|  | Blank | Unknown |
| Primary Purposes | Treatment | Treatment |
|  | Basic Science | Not Treatment |
|  | Device Feasibility |  |
|  | Diagnostic |  |
|  | Health Services Research |  |
|  | Other |  |
|  | Prevention |  |
|  | Screening |  |
|  | Supportive Care |  |
| Phases | Phase 1 | Early Phase |
|  | Phase 1\|2 |  |
|  | Phase 2 |  |
|  | Phase 2\|3 | Late Phase |
|  | Phase 3 |  |
|  | Phase 4 |  |
|  | Blank | Not Applicable |
| Enrollment Count | 1-50 participants | Small |
|  | 51-100 participants | Medium |
|  | 101-500 participants | Large |
|  | 501+ participants | Extra Large |
| IPD Sharing | No | Not Yes |
|  | Undecided |  |
|  | Blank |  |
|  | Yes | Yes |
